# Supplementary material for: Stabilized D2R G protein-coupled receptor oligomers identify multi-state β-arrestin complexes
Source: Nat Commun. 2025 Oct 2;16:8768. doi: 10.1038/s41467-025-64008-7 (PMC12491437; doi:10.1038/s41467-025-64008-7)
Supplement: Supplementary file 1 — Supplementary Information [file 41467_2025_64008_MOESM1_ESM.pdf]

Supplementary Information

Inventory

Supplementary Table 1. Antibodies used in this study

Supplementary Table 2. Plasmids used in this study.

Supplementary Fig. 1. Western blot analysis of HEK 293 cell lysates expressing D2R under non-reducing conditions.

Supplementary Fig. 2. D_2R homomer interactions via BRET and expression levels of D_2R mutants in HEK 293 cells

Supplementary Fig. 3. BRET saturation assays of D2R homomers in HEK293 cells following stimulation with quinpirole.

Supplementary Fig. 4. Models of predicted D_2R homodimers

Supplementary Fig. 5. PD-PALM imaging of cells exhibiting either low or high receptor density to quantify oligomer populations.

Supplementary Fig. 6. Comparison of quinpirole-induced Gαi signaling in either untransfected orD_2R WT expressing HEK293 cells.

Supplementary Fig. 7. βarr2, but not βarr1, is recruited following agonist activation of D_2R WT.

Supplementary Fig. 8. Agonist-induced kinetics of βarr2 recruitment to wildtype (WT) and mutant D_2R.

Supplementary Fig. 9. Lower receptor expression does not induce increased basal βarr2 recruitment or increase either constitutive or ligand-induced internalization

Supplementary Fig. 10. Quinpirole and UNC9994 induce distinct D_2R mediated G-protein signaling and β-arr-2 recruitment profiles.

Supplementary Fig. 11. Plasma membrane localization of WT and mutant D_2R assessed via confocal microscopy.

Supplementary Fig. 12. Quantification of western blots assessing quinpirole-induced ERK1/2 signaling of wildtype and mutant D_2R.

Supplementary Fig. 13. Predicted model for D2R homodimer-Gαi complexes.

Supplementary Fig. 14. BRET saturation assays of D_2R homomers in HEK293 and βarr1/2 knockout cells

Supplementary Fig. 15. Flow cytometry gating strategy from Fig. 4 a-c.

Supplementary Table 1. Antibodies used in this study.

Antibody name	Type	Company	Product number	Dilution
Anti-GAPDH	Primary	Sigma	MAB374	1:1000
HRP-linked horse anti-mouse antibody	Secondary	Cell Signaling	7076	1:2000
M1 Anti- FLAG	Primary	Sigma	3040	1:1000
Alexa Fluor Plus 647 antibody	Secondary	Invitrogen	A32728	1:2000
HRP-linked mouse anti-rabbit	Secondary	Santa- Cruz	sc-2357	1:2000
Anti- Alpha-tubulin	Primary	Cell Signaling	2125	1:1000
Phospho-p44/42 MAPK (erk1/2)	Primary	Cell signaling	9101	1:1000
P44/42 MAPK (erk1/2)	Primary	Cell signaling	9102	1:1000

Supplementary Table 2. Plasmids used in this study.

Plasmid	DNA Construct Source
FLAG-D2R-Rluc8 (wildtype)	Johnathan Javitch, Columbia University
FLAG-D2R-Venus (wildtype)	Johnathan Javitch, Columbia University, Addgene #19966
FLAG- D2R- Rluc8 (V96C)	Michele Poli, Imperial College London
FLAG-D2R-Rluc8 (V96S)	Aylin Hanyaloglu, Imperial College London
FLAG-D2R-Rluc8 (V96S/V97C)	Aylin Hanyaloglu, Imperial College London
FLAG-D2R-Rluc8 (V97C)	Aylin Hanyaloglu, Imperial College London
FLAG-D2R-Rluc8 (Y93C)	Aylin Hanyaloglu, Imperial College London
FLAG-D2R-Venus (V96S)	Aylin Hanyaloglu, Imperial College London
FLAG-D2R-Venus (V96C)	Aylin Hanyaloglu, Imperial College London
FLAG-D2R-Venus (V96S/V97C)	Aylin Hanyaloglu, Imperial College London
FLAG-D2R-Venus (Y93C)	Aylin Hanyaloglu, Imperial College London
β-arrestin2-YFP	Frederic Jean-Alphonse, CNRS, Nouzilly
β-arrestin1-YFP	Frederic Jean-Alphonse, CNRS, Nouzilly

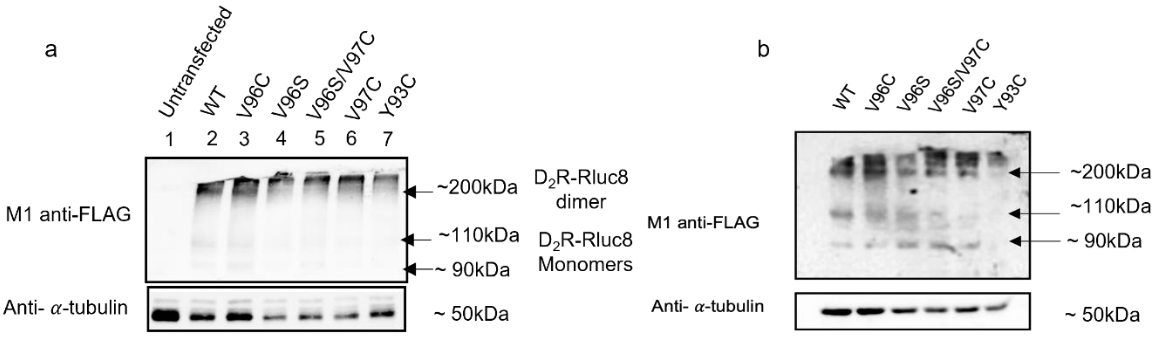

Supplementary Fig. 1. Western blot analysis of HEK 293 cell lysates expressing D_2R under non-reducing conditions. Western blot of HEK293 cell lysates transfected with D_2R-Rluc8 WT or mutant D_2R-Rluc8 as indicated, carried out under non-reducing conditions. These samples originated from the same biological replicate as the Western blot carried out under reducing conditions in Fig. 1 b. Membranes were probed with M1 anti-FLAG antibody to detect FLAG-tagged receptor and anti-α-tubulin was employed as a loading control. Two representative images from different biological replicates (N=4) are shown and were imaged using a chemiluminescence imaging system (**a**) or using X-ray film (**b**).

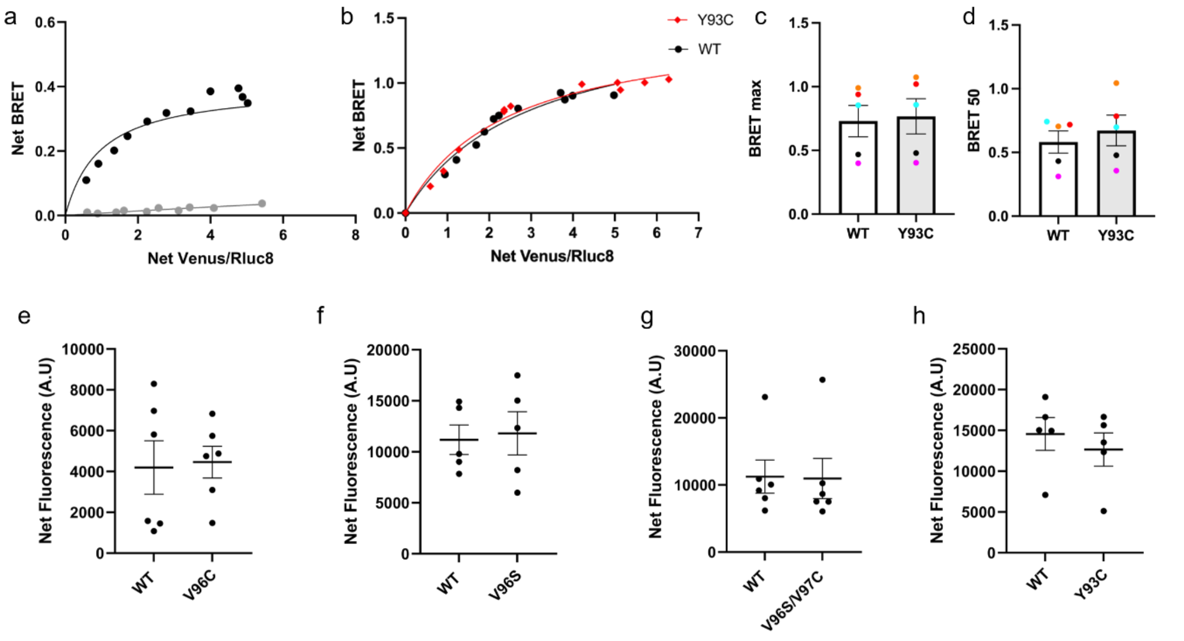

Supplementary Fig. 2. D_2R homomer interactions via BRET and expression levels of D_2R mutants in HEK 293 cells. (a) Representative BRET saturation curve from HEK293 cells were transfected with constant amounts of D_2R-Rluc8 WT with increasing amounts of D_2R-Venus WT plasmid DNA (black). In grey, HEK293 cells transfected with increasing amounts of D_2R-Venus and constant amounts of empty pcDNA3.1_Rluc8 vector plasmid was carried out to demonstrate specificity of saturation obtained in cells co-transfected with D_2R-Rluc8 and D_2R-Venus. (**b**) Representative saturation curves from HEK293 cells transfected with D_2R WT (black) or D_2R Y93C (red) plasmids. Curves used to calculate BRETmax (**c**) or BRET50 (**d**). N=5 independent experiments, mean +/- SEM. Net fluorescence values of D_2R-Venus WT or V96C (**e**), V96S (**f**), V96S/V97C (**g**) or Y93C (**h**) expressing HEK293 cells. Cells were transfected with the same amount of D_2R- Venus plasmid as used in Gi signaling, internalization and pERK assays. N=5 (V96S and Y93C) or 6 (V96C and V96S/V97C) independent experiments, mean +/- SEM. Unpaired, two-tailed Student's t test used to measure statistical differences.

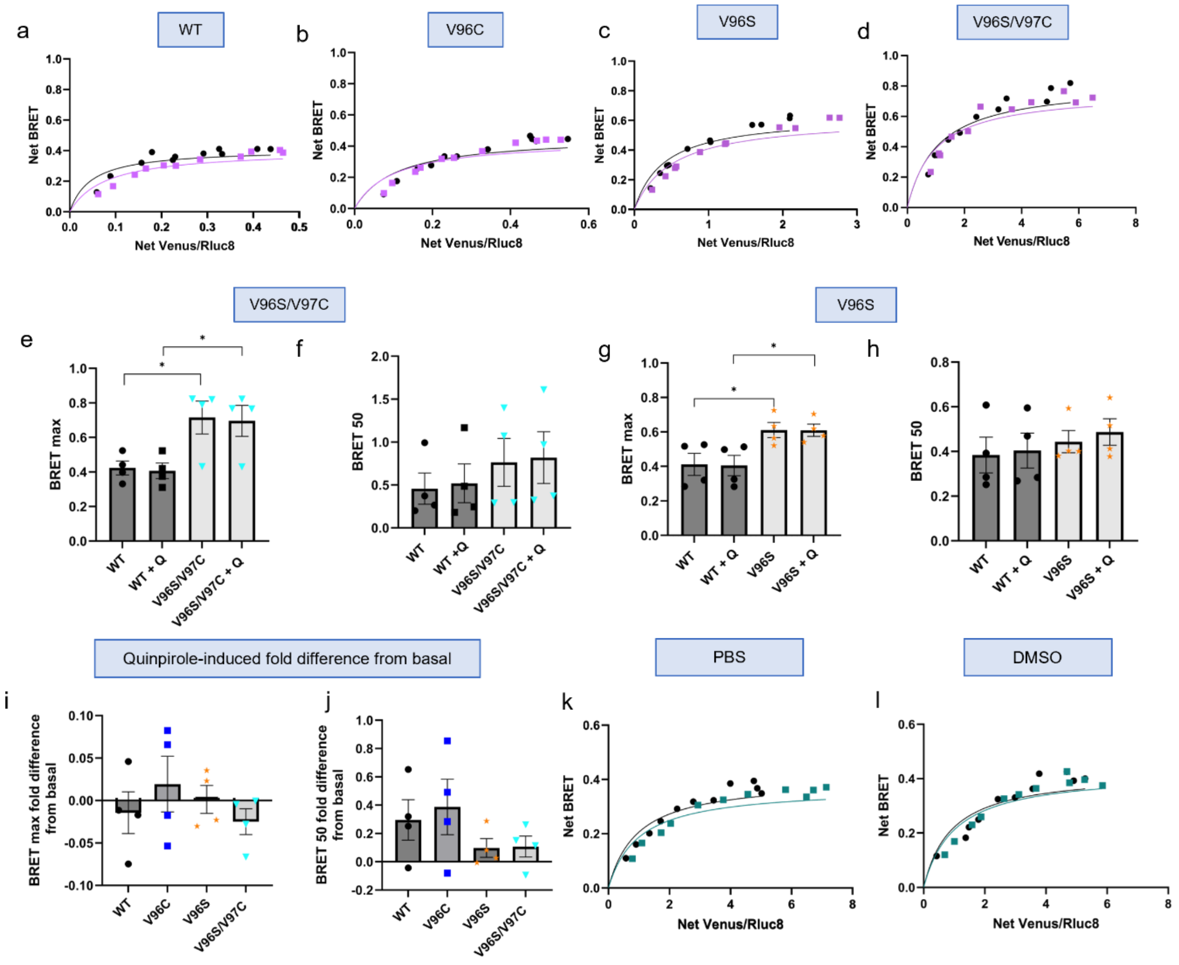

Supplementary Fig. 3. BRET saturation assays of D_2R homomers in HEK293 cells following stimulation with quinpirole. HEK293 cells were transfected with constant amounts of D_2R-Rluc8 WT with increasing amounts of D_2R-Venus WT plasmid DNA (**a and e**) or mutant plasmids V96C (**b**) or V96S (**c**) or V96S/V97C (**d**). BRET signals were acquired before (black) and after addition of 10 µM D_2R agonist quinpirole (purple). Saturation curves were used to quantify BRETmax (**e and g**) and BRET50 (**f and h**) and presented as a fold change from basal in **i** and **j**. N=4, +/- SEM. One-way ANOVA followed by Dunnett's multiple comparison test used to compare basal and quinpirole treated and D_2R WT and mutant expressing cells and across cell lines (**e**) p*=0.042 or 0.0245, (**g**) p*=0.0299 or 0.0281. (**k** and **l**) HEK293 cells were transfected with constant amounts of D_2R-Rluc8 WT with increasing amounts of D_2R-Venus WT plasmid DNA**.** Representative curves show the same transfected cell suspension as in Supplementary Fig.10e and f before (black) and after (green, squares) the addition of PBS (**k**) or DMSO (**l**) as controls.

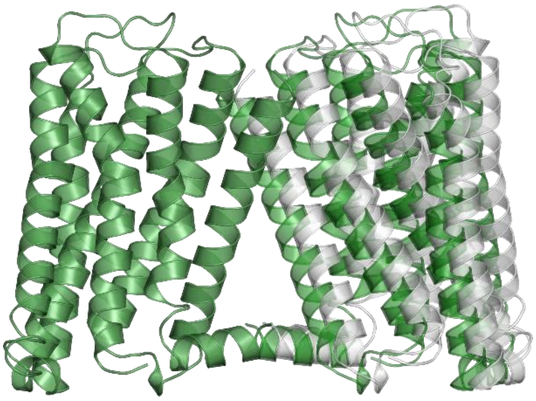

Supplementary Fig. 4. Models of predicted D_2R homodimers. Cartoon representations of the superimposed D_2R homodimers of WT (gray) and V96S/V97C mutant (forest-green) are shown.

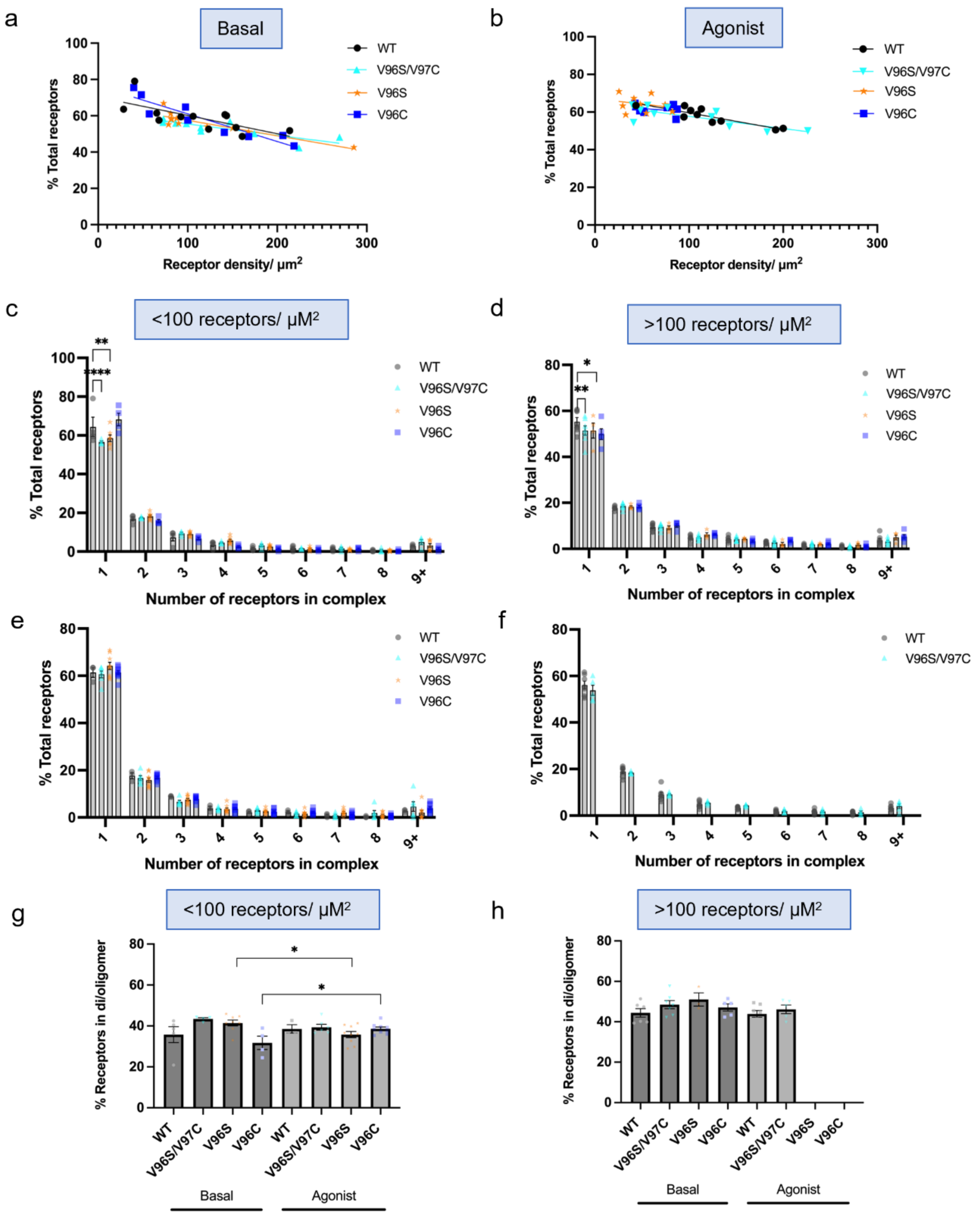

Supplementary Fig. 5. PD-PALM imaging of cells exhibiting either low or high receptor density to quantify oligomer populations. Correlation of D_2R WT or mutant receptor density plotted against proportion of receptors that are not in an oligomeric state (monomers) without agonist stimulation (**a**) or after 5 min of 10 μM quinpirole stimulation (**b**). Composition of D_2R separated into number of receptors in a specific oligomeric complex (1-9 receptor molecules) and expressed as a percentage of total receptors in cells under basal conditions (**c and d**) or after 10 μM quinpirole stimulation **(e and f),** presented as cells with low (**c and e)** or high **(d and f)** receptor density. (**g and h**) Proportion of D_2R WT or mutant receptors as monomers or in di/oligomeric complexes as a percentage of total number of receptors under basal conditions or after 5 min 10 μM quinpirole stimulation separated into cells with low or high receptor density. In c-f two-way ANOVA followed by Šídák's multiple comparisons test used

to measure statistical differences between WT and mutant D_2R in receptor complexes ($p^*=0.047$ (d), $p^{**}=0.0011$ (c) or 0.0013 (d), $p^{****}<0.0001$). In g and h unpaired, two-tailed Student’s t test used to measure differences between WT and mutant D_2R ($p^*= 0.0163$ or 0.0164). N=3 individual experiments, 3-4 cells imaged for each independent repeat.

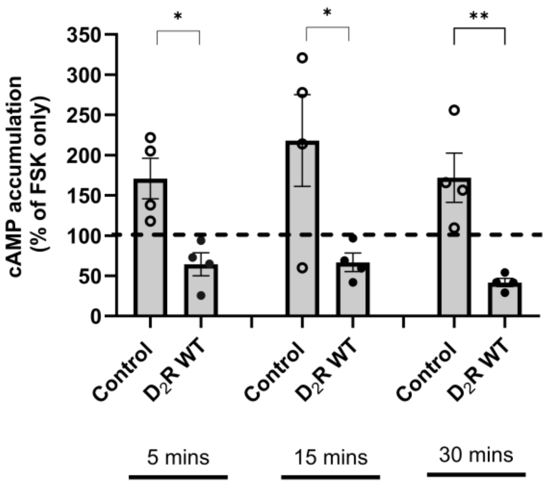

Supplementary Fig. 6. Comparison of quinpirole-induced Gαi signaling in either untransfected or D_2R WT expressing HEK293 cells. cAMP accumulation of HEK293 cells transfected with D_2R WT or untransfected (control) cells measured following 1 μM quinpirole stimulation in the presence of IMBX and forskolin for either 5,15 or 30 min. Graphs represented as a percentage of cAMP levels in cells stimulated with forskolin only (100%), in the absence of agonist. Statistical analysis measuring differences to control untransfected cells within timepoints carried out with unpaired, two-tailed Student's t test (p*=0.0104 or 0.0406, p**= 0.0057). N=4, error bars are +/- SEM.

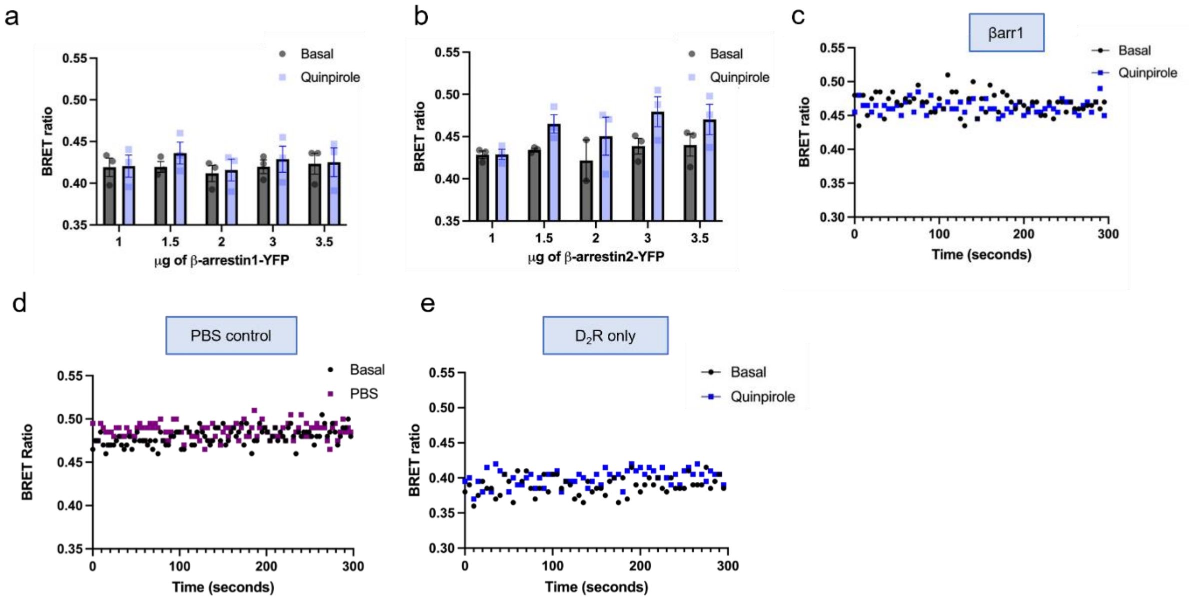

Supplementary Fig. 7. βarr2, but not βarr1, is recruited following agonist activation of D_2R. (**a** and **b**) D_2R-Rluc8 wildtype and increasing amounts of βarr1-YFP (**a**) or βarr2-YFP (**b**) were transfected into HEK293 cells and recruitment to the receptor was assessed before (black) and after (blue) quinpirole agonist addition by measuring BRET ratios. N=3, +/- SEM. (**c**) BRET measurements of cells transfected with D_2R-Rluc8 WT and βarr2-YFP before and after addition of 10 µM quinpirole. (**d**) BRET measurements of HEK293 cells transfected with D_2R-Rluc8 and βarr2-YFP with the addition of PBS (purple) instead of quinpirole after the first measurement, as a control. (**e**) BRET measurements of cells transfected with D_2R-Rluc8 only and no βarr1/2-YFP before and after addition of 10 µM quinpirole. c, d and e show representative curves of at least 4 independent experiments.

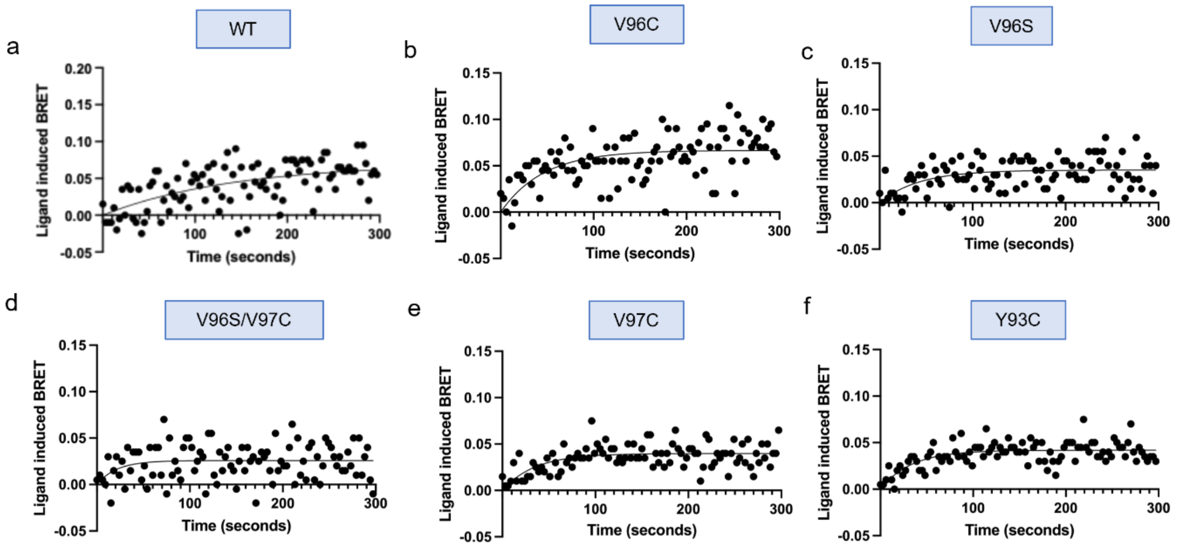

Supplementary Fig. 8. Agonist-induced kinetics of βarr2 recruitment to wildtype (WT) and mutant D_2R. Representative kinetic profile of BRET signals in HEK 293 cells co-transfected with βarr2-YFP and either D_2R-Rluc8 WT (**a**), V96C (**b**), V96S (**c**), V96S/V97C (**d**), V97C (**e**), or Y93C **(f**). Kinetics of curves produced from ligand-induced BRET values are quantified in Table 1. Ligand-induced BRET ratios calculated from BRET values in basal and quinpirole stimulated cells in D_2R transfected cells as shown in Fig. 3. Graphs representative of 4 (Y93C and V97C) or 5 (V96C, V96S and V96S/V97C) independent experiments.

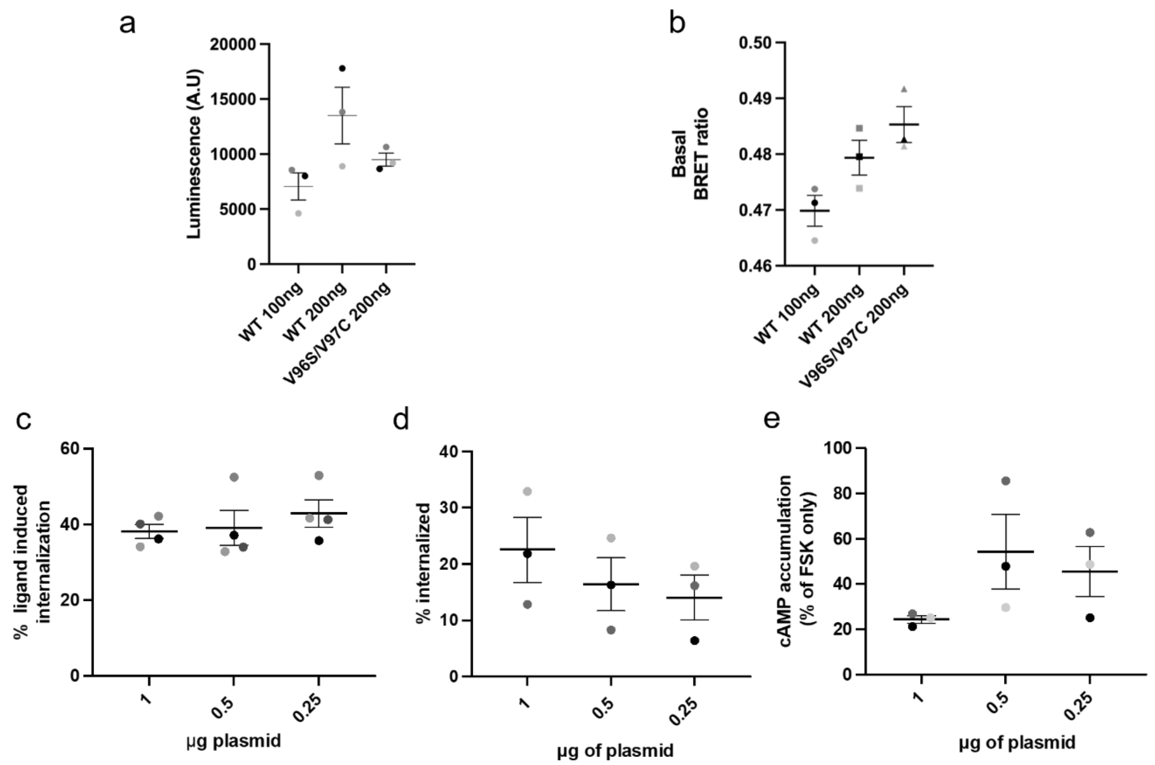

Supplementary Fig. 9. Lower receptor expression does not induce increased basal βarr2 recruitment or, increase either constitutive or ligand-induced internalization. (a) D_2R-Rluc8 luminescence values at 475nm wavelength after addition of coelenterazine-h to quantify receptor expression in HEK293 cells transfected with βarr2-YFP and 100 or 200 ng of D_2R-Rluc8 WT plasmid or 200ng of D_2R-Rluc8 WT plasmid (the equivalent amount of D_2R-Rluc8 plasmid DNA transfected in Fig. 3 and Supplementary Fig. 8). (**b**) Basal BRET values in D_2R-Rluc8 and βarr2-YFP transfected cells, averaged over a time course of 5 min. N=3. Ligand induced internalization (**c**) and constitutive internalization (**d**) of WT D_2R at lower expression levels (comparable to D_2R V96S/V97C expression). Cell surface receptor expression assessed by flow cytometry and % internalization calculated as a decrease in cell surface receptor expression after 10 μM quinpirole stimulation (**c**). For constitutive internalization (**d**), data presented as a percentage difference between cells incubated at 4°C (internalization prevented) and 37°C (internalization can continue). N=3 or 4 (c). (**e**) Forskolin-induced cAMP levels following 5 min 100 nM quinpirole stimulation (taken as maximal response from dose response assays in Fig. 3a), presented as a percentage of cAMP levels in forskolin-only treated cells. Shaded dots correspond to individual biological replicates. N=3, +/- SEM for all experiments.

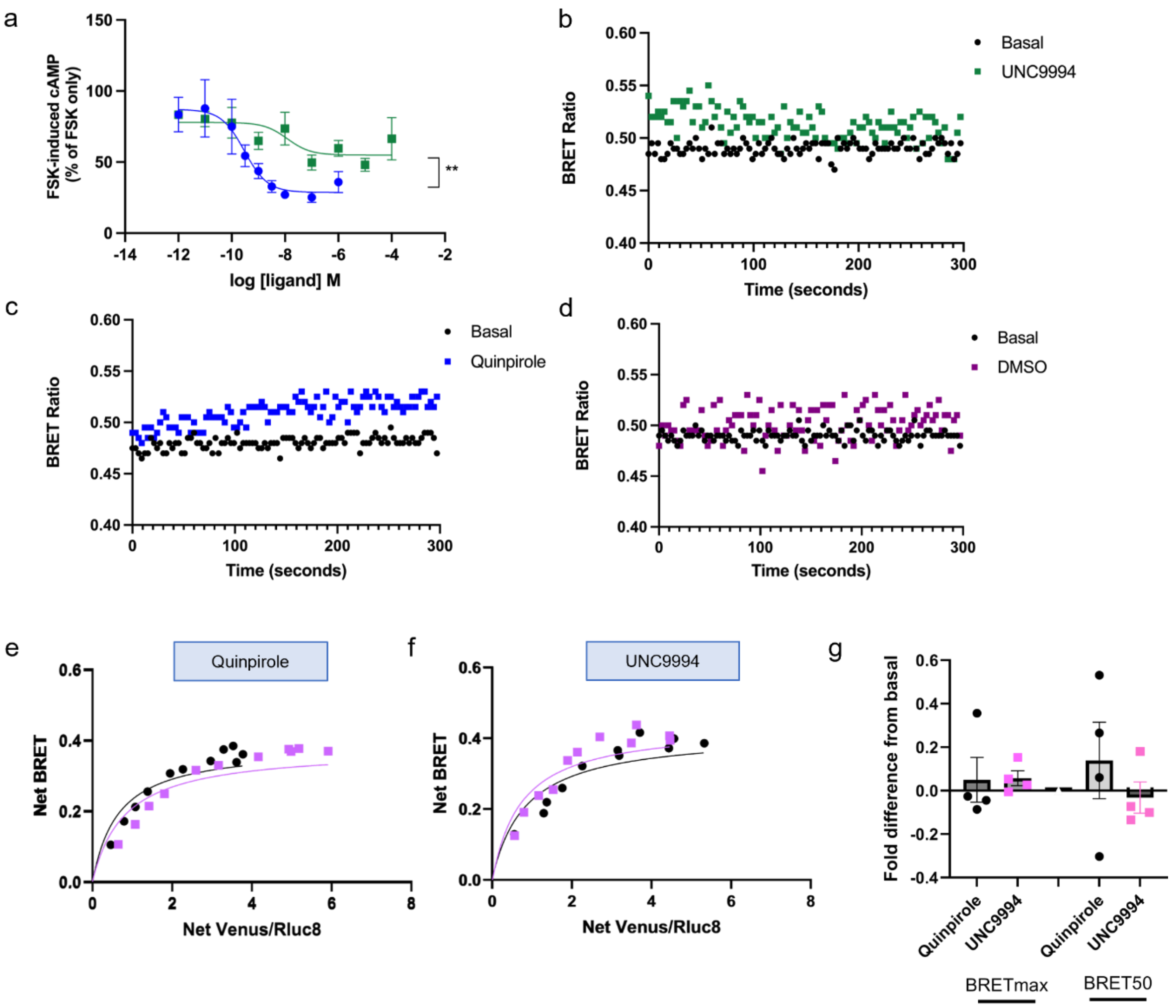

Supplementary Fig. 10. Quinpirole and UNC9994 induce distinct D_2R mediated G-protein signaling and β-arr-2 recruitment profiles. (a) Dose response curves of forskolin-induced cAMP levels in the presence of IBMX in D_2R-Rluc8 WT transfected cells following stimulation with UNC9994 (green) or Quinpirole (blue). N=3, +/- SEM, Statistical significance of differences in maximal Gi response assessed by un-paired Student's t-test, **p= 0.0032. BRET assays showing kinetic profiles of β-arrestin-2 recruitment to D_2R-Rluc8 WT before (black) or after 10 μM quinpirole (blue, **c**) or 10 mM UNC9994 (green, **b**) stimulation or with DMSO instead of ligand (purple, **d**) as a control. Presented as representative profile of 3 independent experiments. (**e-g**) HEK293 cells were transfected with constant amounts of D_2R-Rluc8 WT with increasing amounts of D_2R-Venus WT plasmid DNA**.** Representative saturation curves shown before ligand stimulation (black) or after stimulation with quinpirole (**e**) or UNC9994 biased agonist (**f**). (**g**) Saturation curves used to quantify BRETmax and BRET50 and changes following ligand stimulation presented as a fold difference from basal for each experiment. N=4, mean +/- SEM.

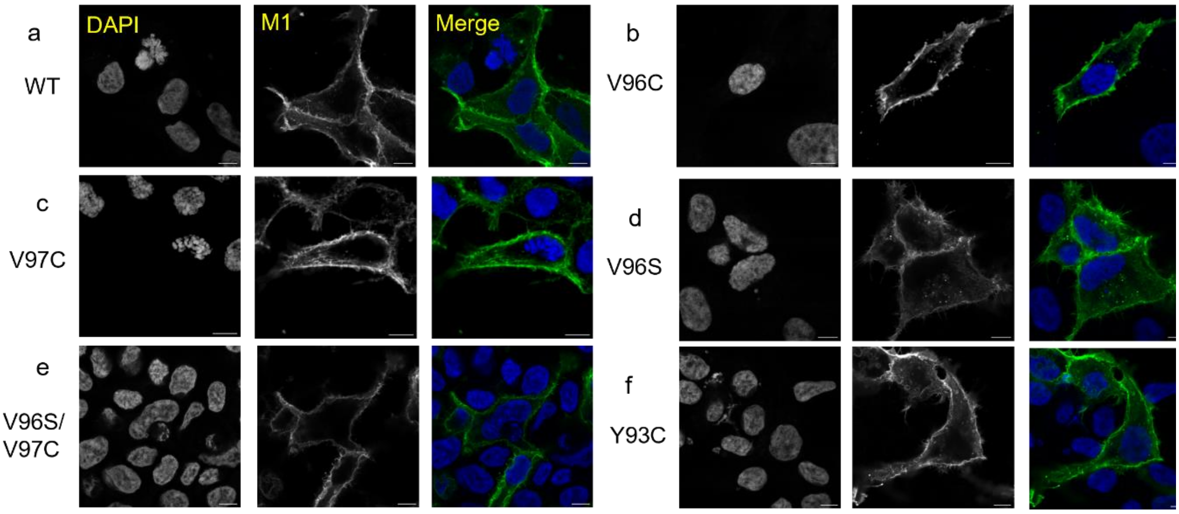

Supplementary Fig. 11. Plasma membrane localization of WT and mutant D$_2$R assessed via confocal microscopy HEK293 cells transiently transfected with N-terminally FLAG tagged (**a**) D$_2$R-Rluc8 WT, (**b**) V96C, (**c**) V97C, (**d**) V96S, (**e**) V96S/V97C, **(f**) Y93C treated with M1 anti-FLAG primary antibody and AlexaFluor647 secondary antibody, fixed and imaged using a confocal microscope to assess plasma membrane expression. Images representative of at least 3 independent experiments. Scale bar= 7 μm.

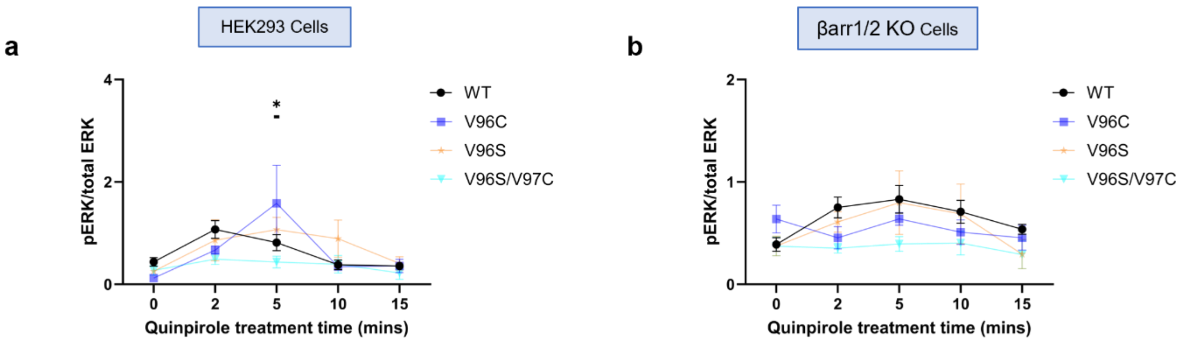

Supplementary Fig. 12. Quantification of western blots assessing quinpirole-induced ERK1/2 signaling of wildtype and mutant D_2R. HEK293 cells (**a**) or HEK293 βarr 1/2 knockout (βarr 1/2 KO) cell lysates (**b**) transfected with D_2R constructs and treated with 10 μM quinpirole for 2, 5, 10 or 15 min, were analysed by western blot and probed with phospho-ERK 1/2 and total ERK 1/2 antibodies. Represented as phospho-ERK1/2/total ERK protein levels. See Fig. 5 for representative western blots and data presented as a fold change from basal. N=8 for D_2R WT expressing HEK293 and β-arr1/2 KO cells, N=6 for D_2R V96C expressed in HEK293 cells, N=5 for D_2R V96S and V96S/V97C expressed in HEK293 cells and D_2R V96S expressed in β-arr1/2 KO cells, N=4 for D_2R V96C and V96S/V97C expressed in β-arr1/2 KO cells. Error bars are +/- SEM. Two-way ANOVA followed by Šídák's multiple comparisons test used to measure statistical differences between WT and mutant D_2R (in a WT vs V96C at 5 min timepoint, p*= 0.0356).

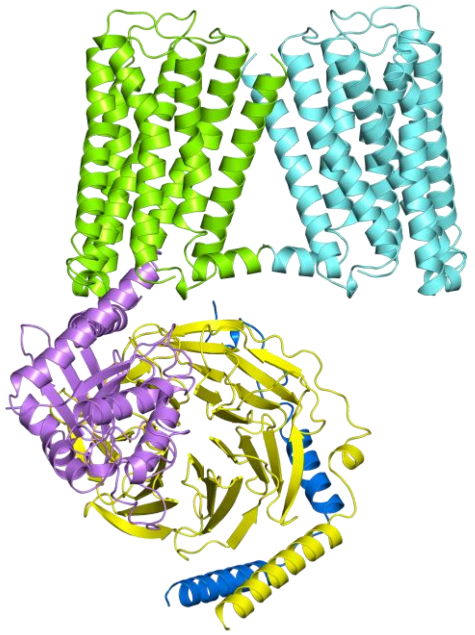

Supplementary Fig. 13. Predicted model for D_2R homodimer-Gαi complexes. The cartoon representation of the predicted D_2R WT homodimer (protomers lemon-green and aquamarine) and heterotrimeric Gi is shown, with a 2:1 receptor: Gi stoichiometry. The α, β, and γ subunits of Gi are shown in violet, yellow, and blue, respectively.

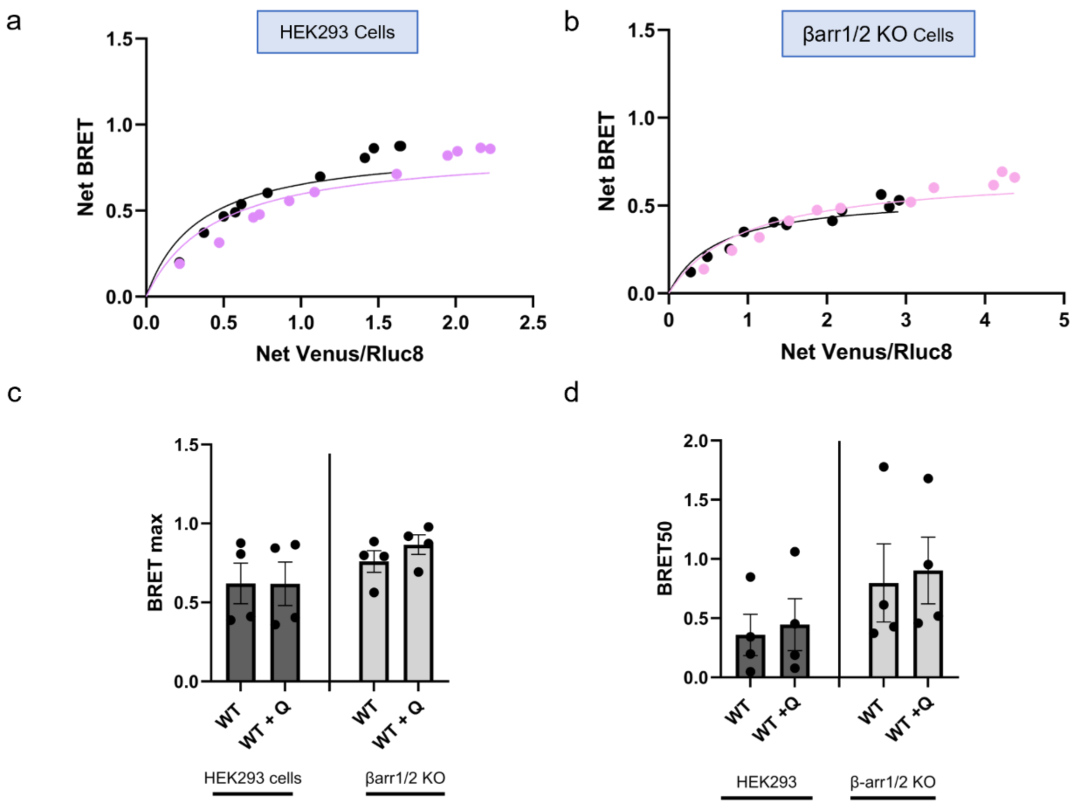

Supplementary Fig. 14. BRET saturation assays of D_2R homomers in HEK293 and βarr1/2 knockout cells. HEK293 cells (**a**) and βarr1/2 knockout cells (**b**) were transfected with constant amounts of D_2R-Rluc8 WT with increasing amounts of D_2R-Venus WT plasmid DNA.BRET measurements were taken before (black) and after addition of 10 μM quinpirole (purple). Saturation curves were used to quantify BRETmax (**c**) and BRET50 (**d**). N=4, +/- SEM, unpaired, two-tailed Student's t test used to measure statistical differences.

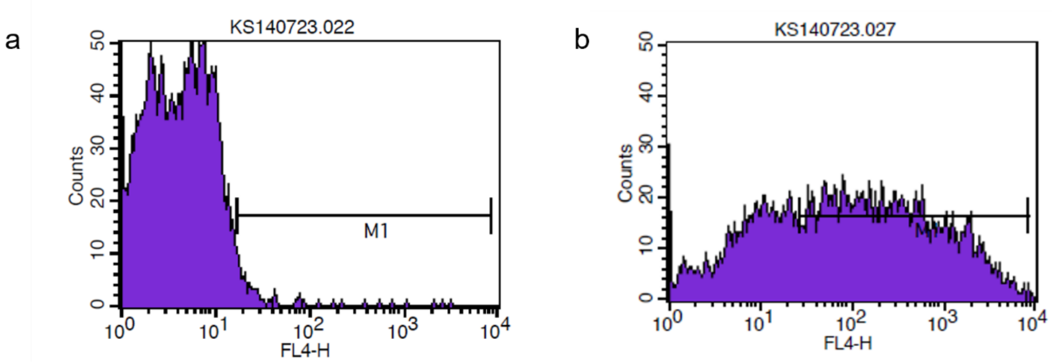

Supplementary Fig. 15. Flow cytometry gating strategy from Fig. 4 a-c. (**a**) Representative gating panel of untransfected HEK293 cells incubated with primary M1 anti-FLAG antibody and secondary AlexaFluor646 antibody and measured by flow cytometry. This was used to determine background and adjust gating accordingly. (**b**) Example gating panel of FLAG-D_2R WT transfected HEK293 cells. Cells within the 'M1' bar were included in analysis.
