# Supplementary material for: Stabilized D2R G protein-coupled receptor oligomers identify multi-state β-arrestin complexes
Source: Nat Commun. 2025 Oct 2;16:8768. doi: 10.1038/s41467-025-64008-7 (PMC12491437; doi:10.1038/s41467-025-64008-7)
Supplement: Supplementary file 2 — Description of Additional Supplementary Files [file 41467_2025_64008_MOESM2_ESM.pdf]

Description of Additional Supplementary Files

File name: Supplementary Data 1
Description: PD-PALM analysis software and scripts.
