# Supplementary material for: Stabilized D2R G protein-coupled receptor oligomers identify multi-state β-arrestin complexes
Source: Nat Commun. 2025 Oct 2;16:8768. doi: 10.1038/s41467-025-64008-7 (PMC12491437; doi:10.1038/s41467-025-64008-7)
Supplement: Supplementary file 4 — Reporting Summary [file 41467_2025_64008_MOESM4_ESM.pdf]

nature portfolio

Corresponding author(s): Aylin Hanyalolgu, Bernadette Byrne

Last updated by author(s): Aug 15, 2025

Reporting Summary

Nature Portfolio wishes to improve the reproducibility of the work that we publish. This form provides structure for consistency and transparency in reporting. For further information on Nature Portfolio policies, see our Editorial Policies and the Editorial Policy Checklist.

Please do not complete any field with "not applicable" or n/a. Refer to the help text for what text to use if an item is not relevant to your study.
For final submission: please carefully check your responses for accuracy; you will not be able to make changes later.

Statistics

For all statistical analyses, confirm that the following items are present in the figure legend, table legend, main text, or Methods section.

n/a	Confirmed	
☐	☒	The exact sample size (n) for each experimental group/condition, given as a discrete number and unit of measurement
☐	☒	A statement on whether measurements were taken from distinct samples or whether the same sample was measured repeatedly
☐	☒	The statistical test(s) used AND whether they are one- or two-sided<br>*Only common tests should be described solely by name; describe more complex techniques in the Methods section.*
☒	☐	A description of all covariates tested
☐	☒	A description of any assumptions or corrections, such as tests of normality and adjustment for multiple comparisons
☐	☒	A full description of the statistical parameters including central tendency (e.g. means) or other basic estimates (e.g. regression coefficient) AND variation (e.g. standard deviation) or associated estimates of uncertainty (e.g. confidence intervals)
☐	☒	For null hypothesis testing, the test statistic (e.g. F, t, r) with confidence intervals, effect sizes, degrees of freedom and P value noted<br>*Give P values as exact values whenever suitable.*
☒	☐	For Bayesian analysis, information on the choice of priors and Markov chain Monte Carlo settings
☒	☐	For hierarchical and complex designs, identification of the appropriate level for tests and full reporting of outcomes
☒	☐	Estimates of effect sizes (e.g. Cohen's d, Pearson's r), indicating how they were calculated

Our web collection on statistics for biologists contains articles on many of the points above.

Software and code

Policy information about availability of computer code

Data collection	BRET assays measured on LUMIstarOPTIMA plate reader (BMG Labtech). Flow cytometry measured using FACS Calibur Flow cytometer (BD biosciences). Confocal microscopy images obtained on a Leica Stellaris 8 microscope. PALM images acquired on Zeiss Eyra PS1 microscope as described in methods.
Data analysis	Most data analysed in GraphPad Prism 10. Western blot bands were analyzed on Image J. PD-PALM analysed in QuickPALM, followed by filtering and neighbourhood analysis using PD-Interpreter as described in methods. The custom code which was used in the analysis of PD-PALM data and is described in the methods section has been attached as Supplementary Data 1. The code is also available on GitHub (

For manuscripts utilizing custom algorithms or software that are central to the research but not yet described in published literature, software must be made available to editors and reviewers. We strongly encourage code deposition in a community repository (e.g. GitHub). See the Nature Portfolio guidelines for submitting code & software for further information.

Data

Policy information about availability of data

All manuscripts must include a data availability statement. This statement should provide the following information, where applicable:

- Accession codes, unique identifiers, or web links for publicly available datasets
- A description of any restrictions on data availability
- For clinical datasets or third party data, please ensure that the statement adheres to our policy

Source data are provided with this paper. Any additional information relating to this study are available from the corresponding author upon reasonable request. Structures used in this study are available on Protein Data Bank: 6CM4 [https://doi.org/10.2210/pdb6CM4/pdb]; 6LUQ [https://doi.org/10.2210/pdb6LUQ/pdb]; 7DFP [https://doi.org/10.2210/pdb7DFP/pdb]; 7JVR [https://doi.org/10.2210/pdb7JVR/pdb]; 8IRS [https://doi.org/10.2210/pdb8IRS/pdb]; 6TKO [https://doi.org/10.2210/pdb6TKO/pdb]; 7SRS [https://doi.org/10.2210/pdb7SRS/pdb]

Research involving human participants, their data, or biological material

Policy information about studies with human participants or human data. See also policy information about sex, gender (identity/presentation), and sexual orientation and race, ethnicity and racism.

Reporting on sex and gender	Human data not used in this study.
Reporting on race, ethnicity, or other socially relevant groupings	Human data not used in this study.
Population characteristics	Human data not used in this study.
Recruitment	Human data not used in this study.
Ethics oversight	Human data not used in this study.

Note that full information on the approval of the study protocol must also be provided in the manuscript.

Field-specific reporting

Please select the one below that is the best fit for your research. If you are not sure, read the appropriate sections before making your selection.

☒ Life sciences ☐ Behavioural & social sciences ☐ Ecological, evolutionary & environmental sciences

For a reference copy of the document with all sections, see nature.com/documents/nr-reporting-summary-flat.pdf

Life sciences study design

All studies must disclose on these points even when the disclosure is negative.

Sample size	All assays were cell based and sample size was determined based on the number required to reliably carry out statistical analysis between groups.
Data exclusions	Some outliers were excluded from analysis but only where it was determined an error had been made (eg transfection unsucessful, loss of cells in a particular condition)
Replication	Number of replicates is stated in individual figure legends and was always at least 3, unless otherwise stated, to enable statistical analysis.
Randomization	Randomization was not required for these cell based assays so is not relevant.
Blinding	Analysis is carried out by computer systems so blinding was not required.

Reporting for specific materials, systems and methods

We require information from authors about some types of materials, experimental systems and methods used in many studies. Here, indicate whether each material, system or method listed is relevant to your study. If you are not sure if a list item applies to your research, read the appropriate section before selecting a response.

Materials & experimental systems

n/a	Involved in the study
☐	☒ Antibodies
☐	☒ Eukaryotic cell lines
☒	☐ Palaeontology and archaeology
☒	☐ Animals and other organisms
☒	☐ Clinical data
☒	☐ Dual use research of concern
☒	☐ Plants

Methods

n/a	Involved in the study
☒	☐ ChIP-seq
☐	☒ Flow cytometry
☒	☐ MRI-based neuroimaging

Antibodies

Antibodies used

Antibodies used in this study are listed in Supplementary Fig. 1.
Anti-GAPDH Primary Sigma MAB374 1:1000
HRP-linked horse anti-mouse antibody Secondary Cell Signaling 7076 1:2000
M1 Anti- FLAG Primary Sigma 3040 1:1000
Alexa Fluor Plus 647 antibody Secondary Invitrogen A32728 1:2000
HRP-linked mouse anti-rabbit Secondary Santa- Cruz sc-2357 1:2000
Anti- Alpha-tubulin Primary Cell Signaling 2125 1:1000
Phospho-p44/42 MAPK (erk1/2) Primary Cell signaling 9101 1:1000
P44/42 MAPK (erk1/2) Primary Cell signaling 9102 1:1000

Validation

Details of the use of all antibodies is provided in the methods section. Antibodies for validated for specificity using appropriate controls as shown in the manuscript figures and methods.

Eukaryotic cell lines

Policy information about cell lines and Sex and Gender in Research

Cell line source(s)

HEK293 cells were purchased from ATCC (CRL-1573).βarr1/2 knockout HEK293 cells were made by using CRISPR-Cas9 (kindly provided by Asuka Inoue, Univ. Tohoku).

Authentication

No further authentication was carried out after purchasing.

Mycoplasma contamination

All cell lines were negative for mycoplasma contamination.

Commonly misidentified lines
(See ICLAC register)

These cell lines are not on the list.

Plants

Seed stocks

Plants not used in this study.

Novel plant genotypes

Plants not used in this study.

Authentication

Plants not used in this study.

Flow Cytometry

Plots

Confirm that:

☐ The axis labels state the marker and fluorochrome used (e.g. CD4-FITC).

☐ The axis scales are clearly visible. Include numbers along axes only for bottom left plot of group (a 'group' is an analysis of identical markers).

☐ All plots are contour plots with outliers or pseudocolor plots.

☒ A numerical value for number of cells or percentage (with statistics) is provided.

Methodology

Sample preparation	Cells were live labelled with M1-anti-FLAG antibody, followed by AlexaFlour647 secondary antibody as described in methods.
Instrument	FACS Calibur Flow Cytometer (BD biosciences)
Software	CellQuest pro built-in software (BD biosciences) followed by GraphPad Prism 10.
Cell population abundance	N/A
Gating strategy	Gated range determined from control unlabelled samples. An example plot is shown in Supplementary Fig. 15.

☒ Tick this box to confirm that a figure exemplifying the gating strategy is provided in the Supplementary Information.

This checklist template is licensed under a Creative Commons Attribution 4.0 International License, which permits use, sharing, adaptation, distribution and reproduction in any medium or format, as long as you give appropriate credit to the original author(s) and the source, provide a link to the Creative Commons license, and indicate if changes were made. The images or other third party material in this article are included in the article's Creative Commons license, unless indicated otherwise in a credit line to the material. If material is not included in the article's Creative Commons license and your intended use is not permitted by statutory regulation or exceeds the permitted use, you will need to obtain permission directly from the copyright holder. To view a copy of this license, visit http://creativecommons.org/licenses/by/4.0/
